# PRO-KIND consensus protocol for classification, monitoring, and therapy in pediatric rheumatology: persistent oligoarticular juvenile idiopathic arthritis

**DOI:** 10.3389/fped.2025.1697137

**Published:** 2026-01-15

**Authors:** Joachim Peitz, Gerd Horneff, Anna Raab, Hanna Winowski, Sandra Hansmann, Klaus Tenbrock

**Affiliations:** 1 Pediatric Rheumatology Center, Asklepios Klinik Sankt Augustin, Sankt Augustin, Germany; 2 Department of Pediatric Rheumatology, Hannover Medical School, Hannover, Germany; 3 Department of Pediatric Rheumatology, St Josef Stift Sendenhorst, Sendenhorst, Germany; 4 Department of Pediatrics, University Hospital Tübingen, Tübingen, Germany; 5 Translational Pediatric Rheumatology and Immunology, RWTH Aachen University Hospital, Aachen, Germany; 6 Department of Pediatrics, Pediatric Rheumatology, University Hospital Bern, Bern, Switzerland

**Keywords:** childhood arthritis, JIA, oligarticular arthritis, recommendations, survey, treatment

## Abstract

Protocols concerning classification, monitoring and treatment were developed for the oligoarticular form of juvenile idiopathic arthritis (JIA) as part of a consensus process. The aim was to establish standardized, evidence-based protocols for managing persistent oligoarticular JIA. The group of authors initially formulated 23 statements and circulated them in an online survey to medical members of the Society for Paediatric and Adolescent Rheumatology (GKJR). A total of 80 of the 124 paediatric and adolescent rheumatologists took part in the survey, which corresponds to just under 65% of the paediatric and adolescent rheumatologists active at the time. In a final online meeting, comments from the survey were incorporated into the statements and then agreed upon by the group of authors. Finally, for newly occurring oligoarticular JIA, 20 statements and a summary consensus treatment protocol were developed to optimise the treatment of persistent oligoarticular JIA.

## Introduction

Persistent oligoarticular JIA is the most commonly diagnosed category of chronic arthritis in children throughout Europe and North America, representing 50%–80% of all pediatric cases. Despite its prevalence, evidence-based guidelines for management remain absent (1). Through the PRO-KIND initiative, protocols for diagnosis, classification, monitoring, and therapy have been developed via an established consensus process.

As part of the Protocols in Pediatric Rheumatology (ProKind) project, protocols for diagnosis, classification, monitoring, and treatment were developed, beginning with polyarticular JIA, according to a previously agreed-upon process (2). The pro-child protocol for the first year of treatment of oligoarticular JIA (ILAR category persistent oligoarthritis) presented here was developed according to this process and is intended to serve as a guide for the indication, implementation, and monitoring of therapy in everyday clinical practice (3). All ProKind protocols are based on existing evidence, the current approval status of drugs, current clinical practice, and previously published guidelines, which are not replaced by these ProKind protocols. Rather, they are intended to harmonize diagnostics, ensure uniform documentation of findings, and promote transparent therapy in order to evaluate the effectiveness of different therapies and thereby optimize the treatment of children with JIA.

The treatment principles already formulated in the guidelines and other protocols for JIA also apply here: “The main goal of therapy is rapid and effective treatment of inflammation with appropriate pain management, control of the underlying disease and, if necessary, induction of remission, prevention of physical disability due to joint contractures, joint destruction, growth disorders in the affected joints resulting in malalignment, preservation of vision, avoiding damage to internal organs, providing support for the psychosocial stress experienced by the patient and their family, and ensuring the largely undisturbed somatic and psychosocial development of children and adolescents”.

The prerequisites for successful treatment of JIA are early diagnosis and referral of patients to physicians with expertise and experience in treating JIA (4). Psychosocial care, awareness of psychosocial comorbidities, non-pharmacological therapy concepts, especially physiotherapy, and a well-prepared, coordinated, and planned transition are established principles in the latest S2k guideline “Treatment of Juvenile Idiopathic Arthritis” (4).

## Definition of oligoarticular JIA

JIA is currently defined and classified according to the International League of Associations for Rheumatology (ILAR) criteria (3, 5). The diagnosis of JIA refers to chronic arthritis that persists for at least six weeks, with onset before the age of 16, after other causes have been ruled out. This means that the patient has chronic arthritis of unknown origin. JIA is subclassified into six diagnostic categories, ultimately only after the first six months of the disease, depending on the number of joints affected and extra-articular manifestations. In persistent oligoarthritis, a maximum of four joints may be affected in the first six months. Exclusion criteria for persistent oligoarticular JIA include, for example, HLA-B27 positivity in boys over the age of 6, the presence of psoriasis, or repeated detection of rheumatoid factors (6, 7). In extended oligoarticular JIA, more than 4 joints are affected over the course of the disease. The same medications are approved for this disease as for polyarticular JIA, so the protocols for polyarticular JIA should be used (2).

In oligoarticular JIA, the main differential diagnoses to be ruled out are infection-associated arthritides, in particular septic arthritis or Lyme arthritis. Reactive arthritis with painful and rapidly occurring arthritis and poor response to nonsteroidal anti-inflammatory drugs may also be a differential diagnosis.

The range of other differential diagnoses includes non-inflammatory causes, especially the consequences of trauma, metabolic diseases (e.g., celiac disease or glycogen storage disorders), hereditary hematological diseases (hemophilia, thalassemia), states of overexertion, but also, in rare cases, oncological diseases such as bone tumors or leukemia.

In the event of additional symptoms such as fever, skin rash, or other organ manifestations, collagenosis or a fever syndrome such as familial Mediterranean fever (FMF) should also be considered in the differential diagnosis.

To date, there are no protocols for the diagnosis, classification, monitoring, and treatment of persistent oligoarticular JIA. The aim of the Pro-Kind project group was therefore to describe current practice patterns with regard to oligoarticular JIA and to develop a consensus-based approach to diagnosis, monitoring, and treatment in the first year of the disease.

## Methods

### Process description for the development of protocols

The creation of the agreed therapy protocols of the Society for Pediatric and Adolescent Rheumatology (GKJR)—in this case, persistent oligoarticular JIA as part of the ProKind project—followed a multi-stage process. Initially, in 2015, at a preparatory meeting to which all members of the GKJR were invited by email, nine different diagnoses were defined as particularly urgent (2, 8).

1. Polyarticular JIA
2. Persistierent oligoarticular JIA
3. Enthesitis associated arthritis EAA-JIA
4. Systemic JIA
5. JIA Uveitis
6. Familiary Mediterranean Fever
7. CASPS/TRAPS/HIDS
8. Systemic Lupus Erythematosus
9. Juvenile Dermatomyositis

Following the preparatory meeting, the GKJR sent out an invitation to all members to actively participate.

During the preparatory meeting, a possible procedure for reaching consensus on protocols was also proposed.

1. Preparation of a draft by at least part of the working group
2. Distribution of the draft to the other participants in the working group
3. Consensus within the working group (consensus with the approval of at least 80% of the participants) by means of a conference call
4. Request for consensus on the draft to all GKJR members in a web survey (again, consensus with the approval of at least 80% of the participants)
5. Consensus on the results within the working group and, if necessary – especially in the case of major changes – submission to all GKJR members in a second web survey
6. Approval of the minutes at a face-to-face online meeting
7. Authorization by the GKJR Executive Board and approval for publication (web and/or print)

The working group for the Pro-Kind Protocol for persistent oligoarticular JIA consisted of six members who had agreed to work on this topic and who are the authors of this article. A total of 23 recommendations were developed based on the available literature on classification, monitoring, and therapy within the working group in three conference calls. As specified in the process described above, all recommendations were adopted by a majority of at least 80% within the working group. Subsequently, all recommendations were sent to all GKJR members for approval as part of a web survey.

## Results

The frequency of agreement among the 80 participants in the web survey is shown in Table 1. 92% of the participants were older than 40 years and 73% were working in an academic or nonacademic hospital. After the results of the web survey were returned, the responses were analyzed and a very high degree of agreement was found overall. Overall, agreement between 87.3% and 100% was achieved. This means that, formally, all statements would have been accepted as agreed upon.

**Table 1:** Approval ratings from the survey of GKJR medical members.

Statement	Approval		Discussion	Disagreement	
	I agree	I agree with additional comment	I disagree, if additional statement is not included	I disagree	Total agreement in %
1	0.97	0.03	0	0	100
2	0.56	0.38	0.05	0.01	93.5
3	0.97	0.01	0.01	0	98.7
4	0.68	0.22	0.08	0.01	90.79
5	0.80	0.11	0.07	0.03	90.79
6	0.85	0.08	0.07	0	93.05
7	0.93	0.03	0.03	0.01	95.94
8	0.61	0.31	0.07	0.01	91.89
9	0.82	0.14	0.03	0.01	98.94
10	0.96	0.03	0	0.01	98.65
11	0.75	0.19	0.06	0	94.44
12	0.85	0.11	0.03	0.01	95.78
13	0.89	0.1	0.01	0	98.61
14	0.86	0.1	0.04	0	95.78
15	0.82	0.1	0.03	0.06	91.66
16	0.83	0.1	0.05	0.01	93.05
17	0.77	0.1	0.08	0.04	**87** **.** **32**
18	0.87	0.08	0.04	0	95.83
19	0.87	0.08	0.03	0.01	95.77
20	0.93	0.06	0	0.01	98.62
21	0.87	0.07	0.01	0.04	94.36
22	0.83	0.08	0.06	0.03	91.55
23	0.66	0.25	0.07	0.01	91.55

The first two response options were formulated in such a way that they were evaluated as unconditional agreement. However, the second response option allowed for the possibility of suggesting a change. Response options 3 and 4 indicated disagreement with the statement, with the third response option making agreement contingent upon a change to the statement. The selection of the first two response options was defined as agreement overall.

Repeatedly expressed suggestions for change from the second and third response options were compiled. Proposed changes were discussed in the working group and, insofar as they altered the statements, also recorded. This took place during a face-to-face meeting. As a result of the working group meeting, two of six related statements were summarized, resulting in a total of 20 statements being agreed upon.

The statements corresponding to the numbers in the final version are formulated as follows:

## General statements on diagnosis

The following statements on diagnosis, monitoring, and treatment apply to confirmed persistent oligoarticular JIA. In order to diagnose oligoarticular JIA, the patient must have had arthritis in fewer than four joints for at least six weeks.

**Table:** 

**Statement 1:** The consensus treatment protocol is intended to apply to the following ILAR-defined JIA category (inclusion criteria): persistent oligoarticular course. The consensus treatment protocol should not apply to (exclusion criteria): any other form [systemic JIA, psoriatic arthritis, enthesitis-associated (EAA) JIA, rheumatoid factor-positive or -negative polyarticular JIA, extended oligoarticular JIA]. (100%)
**Statement 2**: The minimum basic diagnostics required for diagnosis and differential diagnosis should include: complete blood count including differential blood count, ESR, CRP, ASAT, ALAT, GGT, creatinine, CK, LDH, AP, urine status, ANA, HLA-B27, celiac serology. If there are indications of a previous infection or gastrointestinal symptoms, further targeted diagnostics should be performed if necessary (e.g., Borrelia serology, mycoplasma, Yersinia, other reactive pathogens, or calprotectin in stool). (A) (90.7%)

Comments on statement 2:

A: Statement 2 was summarized from statements 2 and 4 based on comments from the web survey:

Web survey statement 2: The minimum basic diagnostics required for diagnosis and differential diagnosis should include: blood count including differential blood count, ESR, CRP, ASAT, ALAT, GGT, creatinine, uric acid, CK, LDH, AP, urine status, RF, HLA-B27, ANA, IgG, IgA, IgM, Borrelia serology

Web survey statement 4: If there are indications of a previous infection or gastrointestinal symptoms, further targeted diagnostics should be performed (ASL, mycoplasma, Yersinia or HP antigen and calprotectin in stool, celiac disease AK in serum).

## Statements on diagnostic and monitoring

**Table:** 

**Statement 3:** In every patient, uveitis should be ruled out promptly, ideally within 2 weeks. All children with oligoarticular JIA should undergo regular ophthalmological examinations to rule out uveitis, initially at 3-month intervals, in accordance with the published AMWF guideline (registration number 045–012). (B) (94.36%)
**Statement 4:** Before starting immunosuppressive therapy, vaccination antibodies (VZV, measles, HepB) should be determined if vaccination status is uncertain, and efforts should be made to complete the vaccination status.(90.79%)
**Statement 5:** Imaging diagnostics should include an ultrasound examination of at least all clinically affected joints. Further imaging should be performed in unclear cases for differential diagnosis or if temporomandibular joint involvement or cervical spine involvement is suspected, e.g., x-ray, MRI. (93.95%)
**Statement 6:** The Juvenile Arthritis Disease Activity Score-10 (JADAS-10) or the clinical JADAS-10 and the Childhood Health Assessment Questionnaire (CHAQ) can be used to assess disease activity and functional impairment. (C) (95.94%)
**Statement 7:** Definition of safety parameters depending on activity status and therapy: The following should be performed during follow-up examinations: Complete blood count including differential blood count, ESR, CRP, creatinine, ALAT, GGT, LDH, urine status (91.89%)
**Statement 8:** Clinical check-ups during the first year of treatment should be carried out at intervals of no more than 4–6 weeks until improvement is seen, then every 3 months. (98.94%)
**Statement 9:** Definition of the goals of drug therapy for the first year of treatment: JADAS inactive disease (JADAS10 ≤1.4) is the actual goal, JADAS MDA (minimal disease activity, JADAS10 1.4 to ≤4) is an “acceptable” goal, and JADAS10 > 4 corresponds to unacceptable disease activity. Preventing joint damage is another treatment goal. (98.65%)

Comments on statements 3–9:

B: Statements 3 and 21 on uveitis have been combined:

Web survey statement 3: Uveitis should be ruled out promptly in every patient, ideally within 2 weeks. Web survey statement 21: All children with oligoarticular JIA should undergo regular ophthalmological examinations to rule out uveitis, initially at 3-month intervals, in accordance with the published AMWF guideline (registration number 045–012).

C: At the time of the consensus conference, the new JADAS criteria had not yet been published. These have now been updated and included in the statement (7). A JADAS-10 score of ≤1.4 corresponds to inactive disease, a JADAS-10 score of 1.5 to ≤4 corresponds to minimal disease activity, a JADAS-10 score of 4.1 to ≤13 corresponds to moderate disease activity, and a JADAS-10 score >13 corresponds to high disease activity. For the cJADAS, the thresholds are ≤1.1; 1.2–4; 4.1–12 and >12.

## General therapy statements

**Table:** 

**Statement 10:** Symptomatic treatment with nonsteroidal anti-inflammatory drugs should be administered at the usual dosages, if possible within the scope of the approval, if the corresponding symptoms are present (see Table 2). (94.44%)
**Statement 11:** The use of NSAIDs alone may be sufficient in cases of self-limiting disease lasting only a few weeks or months. In cases of active arthritis lasting longer than 4 weeks with pain and/or impairing movement restrictions, treatment with NSAIDs alone is inadequate. (D) (95.78%)
**Statement 12:** Intra-articular therapy with corticosteroids may be indicated for any active arthritis. This may be an initial component of therapy or may be administered in addition to other therapeutic measures during the course of treatment. If treatment fails, intra-articular therapy with corticosteroids should generally not be repeated until 3 months after the last injection. In individual cases, prompt repetition of the injection may be considered if there is no effect. (E) (91.66%)
**Statement 13:** Triamcinolone hexacetonide (TH) is preferable to other preparations. Dosage for TH: 0.5–1 mg/kg body weight in large joints, max. 40 mg/joint (knee, hip, shoulder), up to 0.5 mg/kg body weight in medium-sized joints (hand, ankle, elbow joints), max. 20 mg/joint, and up to max. 2 mg per small joint (fingers, toes). (F) (95.78%)
**Statement 14:** Systemic therapy with methotrexate (MTX) (10–15 mg/m^2^ body surface area once a week) may be indicated for any active arthritis. This may be an initial component of therapy or may be added to other therapeutic measures during the course of treatment, but it should be checked whether a transfer to another Pro-Kind protocol appears necessary. MTX is not approved for oligoarticular JIA. (G) (93.05%)
**Statement 15:** Systemic therapy with sulfasalazine (50 mg/kg body weight, max. 2 g/day) may be indicated for any active arthritis—after exhausting approved therapies—especially if HLA-B27 is detected. This may represent an initial therapeutic component or be administered in addition to other therapeutic measures during the course of treatment. In any case, the diagnosis should be reviewed and, if necessary, transfer to another pro-child protocol should be considered. Sulfasalazine is not approved for oligoarticular JIA. (H) (87.32%)
**Statement 16:** Therapy failure can be defined as a lack of improvement in JADAS. Furthermore, therapy failure can be determined if, after a therapy period of at least 6 months, the threshold for acceptable disease activity has not been reached. (95,83%)
**Statement 17:** Furthermore, treatment failure can be determined if, after a treatment period of at least 12 months, the threshold for minimal disease activity (JADAS10 < 4) has not been reached. (95,77%)
**Statement 18:** In cases of insufficient response/treatment failure to adequate therapy with intra-articular steroids and NSAIDs, combination therapy with MTX and intra-articular therapy with corticosteroids may be appropriate. (98,62%)
**Statement 19:** In cases of insufficient response/treatment failure, an extension of therapy with biologics or small molecules (TNF blockade, CTLA4 blockade, IL-6 blockade, JAKi) may be considered. This constitutes off-label use for oligoarticular JIA. Adalimumab is only approved for uveitis. In this case, it should be checked whether a transfer to another Pro-Kind protocol appears necessary. (I) (91,55%)
**Statement 20:** In the event of remission during treatment, systemic therapy (MTX) should be continued for at least 12 months; in individual cases, earlier reduction of therapy may be appropriate. (91.55%)

Comments on statements 10–20:

**Table 2:** Dosage of nonsteroidal anti-inflammatory drugs in the treatment of oligoarticular JIA.

Medication	Daily dosage	Remarks
Naproxen	10–15 mg/kgBW in 2 SD	Age limit >1 year. Juice formulation available.
Ibuprofen	30–40 mg/kgBW in 3–4 SD	Approved for children aged 6 months and older, juice formulation available
Indometacin	2–3 mg/kgBW in 3–4 SD	Approved for children aged 2 years and older, juice formulation available
Diclofenac	2–3 mg/kgBW in 2–3 SD	Approved for children aged 9 years and older
Meloxicam	0.25–0.375 mg/kgBW in 1 SD	(Not approved for JIA, approved for rheumatoid arthritis, ankylosing spondylitis, and osteoarthritis in patients aged 16 years and older)
Celecoxib	6–12 mg/kgBW in 2SD	(Not approved for children and adolescents in Germany; approved in the US for children aged 2 years and older)

D: At the time of diagnosis and entry into force of this statement, active arthritis has already been present for 6 weeks. In the case of insufficient therapy with NSAIDs up to that point, or if improvement has occurred at the time of diagnosis, therapy with NSAIDs alone may be sufficient for a further 4 weeks. However, if active arthritis persists beyond this period, therapy should be extended.

E: Statement 12 was summarized from statements 13 and 15 of the web survey after comments.

Web survey statement 13: Intra-articular therapy with corticosteroids may be indicated for any active arthritis. This can be an initial component of therapy or can be administered in addition to other therapeutic measures during the course of treatment. The therapy can be repeated at intervals of several months.

Web survey statement 15: If therapy fails, intra-articular therapy with corticosteroids should not generally be repeated until 3 months after the last injection.

F: (9, 10).

G: (11, 12).

H: (13).

I: A corresponding protocol would be polyarticular JIA or EAA (2, 18) At the time of the consensus, JAK inhibitors were not yet approved for polyarticular JIA; approval has since been granted for certain categories of JIA (14). The authors have now supplemented these.

A recommendation for off-label use is expressly not made. However, off-label therapy may be indicated in the opinion of the treating physician and is possible if a serious health impairment or painful condition cannot be effectively treated due to a lack of therapeutic alternatives. In addition, research results must be available that suggest that the drug could be approved for the indication in question.

**Figure 1:**
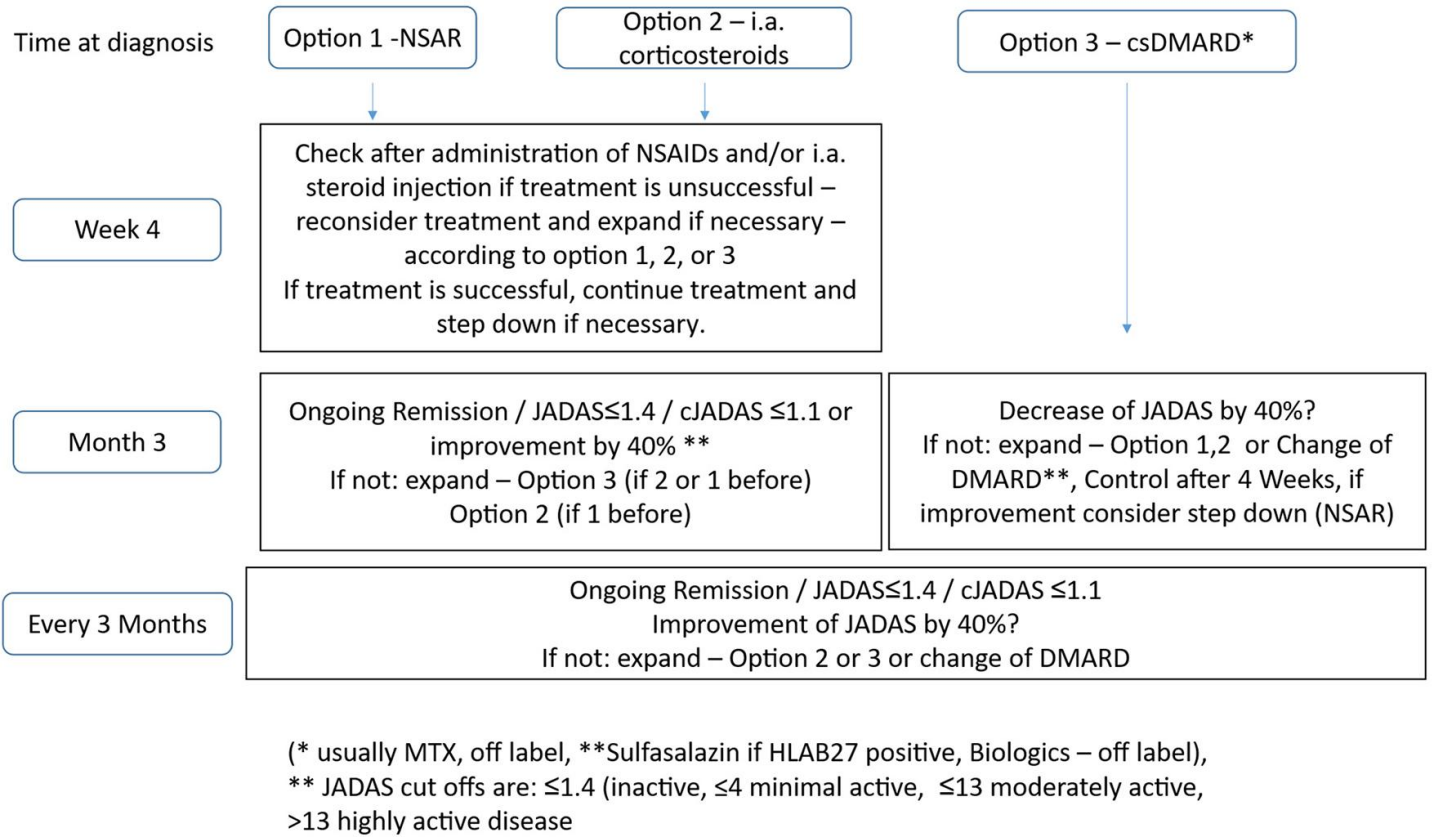
Consensus treatment protocol for oligoarticular JIA (without uveitis): there are a total of three equivalent treatment options available, which are used depending on the severity of the findings (how severely the patient is affected) and the preferences of the family and the treatment center. They can be used individually or in combination. Except when starting treatment with a DMARD (option 3) with a delayed onset of action, an initial follow-up interval of 4 weeks appears reasonable for assessing the response to treatment. Regular check-ups should be carried out every 3 months for all treatment options. Systemic glucocorticoids can be used in cases of high disease activity. Long-term use should be avoided due to undesirable effects and the availability of other forms of therapy (see AWMF guideline on JIA, Oommen et al.). The goal is remission of the disease after 12 months at the latest. If the disease progresses to extended oligoarticular JIA, switching to the protocol for polyarticular JIA is recommended. NSAIDs, non-steroidal anti-inflammatory drugs; DMARDs, disease-modifying anti-rheumatic drugs; disease-modifying basic therapeutics; MTX, methotrexate.

The statements on therapy were summarized by the working group in a therapy algorithm (Figure 1).

## Discussion

These are the first protocols for the diagnosis, classification, monitoring, and treatment of persistent oligoarticular JIA. Using a previously agreed method, 23 recommendations were initially developed and, following the survey, summarized and agreed upon as a total of 20 statements. The consensus recommendations presented here are explicitly not comprehensive guidelines, but primarily serve to harmonize the diagnosis, treatment, and monitoring of the disease. The idea behind this ProKind process is that this harmonization will lead to an improvement in treatment in the sense of treat-to-target with an improvement in outcome. However, limitations are that the recommendations are based on the situation in the German healthcare system. Moreover, 35% of German pediatric rheumatologists did not take part in the survey.

Moreover, these recommendations for action are also limited by the lack of randomized clinical trials, with the exception of the study by Ravelli et al. (12) and of Hissink-Mueller, in which also orligarticular patients participate (15). Moreover our recommendations are hampered by the resulting lack of evidence for the efficacy of the various drugs used in off-label therapy. Understanding of the pathogenesis and the increasing number of treatment options have also influenced the goals of treating chronic inflammatory rheumatic joint diseases in children (16).

Today, low disease activity and, if possible, inactive disease are realistic treatment goals (6, 7). The available treatment protocols are suitable for making this achievable for as many patients as possible. This has already been demonstrated in a treat-to-target study for polyarticular JIA (17).

The evaluation of data from more than 350 oligoarticular patients included in the ProKind Rheuma (Treat to Target) project funded by the Joint Federal Committee (GBA) and led by the last author of this article also suggests this (publication in preparation). The project examines the application and outcome of the Treat to Target approach. The authors of this consensus article hope that the protocols and the Treat to Target approach will become widely accepted in everyday clinical practice.
